# Associations between sheep meat intake frequency and blood plasma levels of metabolites and lipoproteins in healthy Uzbek adults

**DOI:** 10.1007/s11306-023-02005-x

**Published:** 2023-04-26

**Authors:** Diyora Kurmaeva, Yongxin Ye, Inal Bakhytkyzy, Violetta Aru, Dilbar Dalimova, Shahlo Turdikulova, Lars Ove Dragsted, Søren Balling Engelsen, Bekzod Khakimov

**Affiliations:** 1 Centre for Advanced Technologies, Talabalar Shaharchasi 3A, 100041 Tashkent, Uzbekistan; 2 Department of Food Science, University of Copenhagen, Rolighedsvej 26, 1958 Frederiksberg C, Denmark; 3 Department of Nutrition, Exercise and Sports, Faculty of Science, University of Copenhagen, Rolighedsvej 26, 1958 Frederiksberg, Denmark

**Keywords:** Meat intake, Sheep meat, NMR, Metabolomics, Lipoproteins, Choline

## Abstract

**Introduction:**

Uzbekistan is one of the countries with the highest number of diet-related chronic diseases, which is believed to be associated with high animal fat intake. Sheep meat is high in fats (~ 5% in muscle), including saturated and monounsaturated fatty acids, and it contains nearly twice the higher amounts of *n-3* polyunsaturated fatty acids and conjugated linoleic acids compared to beef. Nevertheless, sheep meat is considered health promoting by the locals in Uzbekistan and it accounts for around 1/3 of red meat intake in the country.

**Objectives:**

The aim of this study was to apply a metabolomics approach to investigate if sheep meat intake frequency (SMIF) is associated with alterations in fasting blood plasma metabolites and lipoproteins in healthy Uzbek adults.

**Methods:**

The study included 263 subjects, 149 females and 114 males. For each subject a food intake questionnaire, including SMIF, was recorded and fasting blood plasma samples were collected for metabolomics. Blood plasma metabolites and lipoprotein concentrations were determined using ^1^H NMR spectroscopy.

**Results and Conclusion:**

The results showed that SMIF was confounded by nationality, sex, body mass index (BMI), age, intake frequency of total meat and fish in ascending order (*p* < 0.01). Multivariate and univariate data analyses showed differences in the levels of plasma metabolites and lipoproteins with respect to SMIF. The effect of SMIF after statistical adjustment by nationality, sex, BMI, age, intake frequency of total meat and fish decreased but remained significant. Pyruvic acid, phenylalanine, ornithine, and acetic acid remained significantly lower in the high SMIF group, whereas choline, asparagine, and dimethylglycine showed an increasing trend. Levels of cholesterol, apolipoprotein A1, as well as low- and high-density lipoprotein subfractions all displayed a decreasing trend with increased SMIF although the difference were not significant after FDR correction.

**Supplementary Information:**

The online version contains supplementary material available at 10.1007/s11306-023-02005-x.

## Introduction

Historically, meat has played an important role in human evolution, and until now it remains an important part of our diet providing essential nutrients (Pereira & Vicente, 2013). Meat provides key nutrients such as essential amino acids, some beneficial fatty acids, vitamins, and minerals. Of the above-mentioned nutrients, some are necessary to support the human bodily functions, such as protein turnover, which is maintained by essential amino acids (Williams, 2007). Differently, an excessive dietary consumption of some fatty acids, especially saturated and trans fatty acids, has been associated with an increased risk of cardiovascular disease. Therefore, red meat and animal fat have been extensively investigated for their fatty acid profiles, which may vary depending on the type of meat, the age of animals, geographical location, and feeding regime (Hou et al., 2020; Ortiz et al., 2021). Meat sources are normally classified into white meat (poultry and aquatic products) and red meat (pork, beef, mutton and lamb), but different types of red meats have different fatty acid profiles, nutritional value and are thus expected to vary in their influence on human health. Among red meats, sheep meat, including mutton and lamb, contains higher levels of heme iron (h-Fe) compared to beef (Pretorius et al., 2016). H-Fe is more bioavailable, i.e. easier for humans to absorb from the diet, compared to non-heme iron (Ponnampalam et al., 2016). Apart from iron, sheep meat is a rich source of other minerals, such as Ca, Mg, Co, Cu and Se, as well as vitamins like vitamin A, vitamin B12 and vitamin E (Ponnampalam et al., 2016). Like in other red meats, sheep meat contains relatively similar amounts of saturated fatty acids, including C16:0, C18:0 and C14:0, and monounsaturated fatty acids (mainly C18:1) that constitute the major proportion of fats in meat, while trans- and polyunsaturated fatty acids constitute a comparatively minor proportion of the fat components (Wyness et al., 2011).

Sheep muscle contains relatively higher amounts of *n-3* polyunsaturated fatty acids (PUFA) (2.49 ± 0.99%) compared to beef muscle (1.48 ± 0.02%) while the content of *n-6* PUFA is similar, 3.34 ± 1.28% and 3.05 ± 0.06%, respectively (Chikwanha et al., 2018). Likewise, sheep muscle contains slightly higher levels of conjugated linoleic acids (CLA) (10.3, ± 5.2%), total saturated fatty acids (48.3, ± 2.09%), and total *trans* monounsaturated fatty acids (MUFA) (6.2, ± 1.79%) than beef, where the relative concentrations of these fatty acids are 4.9 (± 1.8%), 43 (± 1.7%), and 3.8 (± 0.23%), respectively. However, the level of total *cis* MUFA is lower in sheep muscle (32.5, ± 4.83%) than in beef (49.9, ± 1.84%). Even though a high dietary intake of saturated fatty acids has been linked to an increased level of low-density lipoprotein (LDL, “bad”) cholesterol, a previous study showed that they do not decrease high-density lipoprotein (HDL, “good”) cholesterol and thus claimed not to contribute to the development of arterial plaques as much as for example *trans* fats (Siri-Tarino et al., 2010). Some animal and human studies have reported associations between CLA and reducing the risk of cardiovascular diseases, diabetes, cancer, and obesity (Basak & Duttaroy, 2020; Fuke & Nornberg, 2017). For populations in Uzbekistan, who consume less fish than many other countries in the world, lean sheep meat can be considered as a complementary source of *n* − 3 PUFA (Ponnampalam et al., 2021), providing up to 60 mg of this beneficial fatty acid per 100 g of meat (Ponnampalam et al., 2016). Despite being an important ingredient in traditional cuisine around the world, the effect of sheep meat intake on the human metabolomes has not been investigated yet. Differently, the impact of pork (Wade et al., 2019) or beef (Bertram & Jakobsen, 2018) intake on human health and metabolomes has been investigated in a few studies.

In Uzbekistan, sheep meat is considered health promoting by the locals and accounts for around 1/3 of red meat intake in the country. We hypothesise that people who consume sheep meat more frequently, thus less beef or other red meat types, have distinct fasting blood plasma metabolome and lipoprotein profiles than those who do not or consume less, which might be attributed to less blood plasma levels of LDL cholesterol. Thus, this study aims to investigate if sheep meat intake frequency (SMIF) is related to distinct changes in human blood plasma metabolome and/or lipoproteins in healthy Uzbek adults. To do so, a total of 263 healthy subjects were recruited amongst the Uzbek population, which were divided into three groups: high SMIF (> 10 times per month), moderate (1–10 times per month) and zero (less than 1 time per month) SMIF. Fasting blood plasma was collected from each individual and measured by proton (^1^H) nuclear magnetic resonance (NMR) spectroscopy to determine plasma levels of metabolites and lipoproteins (Khakimov et al., 2022; Monsonis Centelles et al., 2017). This work describes the levels of individual metabolites and lipoproteins between the three groups of subjects stratified according to SMIF, high, moderate or zero SMIF, which will provide a theoretical basis for the understanding of the metabolome and lipoprotein changes related to sheep meat consumption.

## Materials and methods

### Subjects

A total of 263 healthy subjects, 149 females and 114 males, were included in the study and represented subjects between age of 20 to 85 years old and body mass index (BMI, kg/m^2^) of 16.8 to 40. Exclusion criteria were pregnant or lactating women (up to 6 weeks before the start of the study), the subjects who were diagnosed with any form of cardiovascular disease or diabetes, cancer, reporting chronic gastrointestinal disorders, or those receiving lipid-lowering, anti-diabetic, or anti-hypertensive drugs, including preventive medications, as well as antibiotic treatment within the last three months before the start of study. All subjects were recruited at Center for Advanced Technologies (Tashkent, Uzbekistan) and provided a written consent to participate in the study. The study was in accordance with the Helsinki declaration and approved by a local Ethical Committee (ECDL-01–01092018) at the Center for Advanced Technologies of the Republic of Uzbekistan. Most of the subjects were recruited from three research and educational institutions, and two food production plants, thus represented researchers, teachers, students, and factory workers with middle to high educational level and an average income. Nearly 90% of the individuals were ethnic Uzbeks and the remaining were ethnic Russians, Tatars, or other minorities. The demographic information and lifestyle, including BMI (kg/m^2^), smoking status and alcohol consumption (monthly frequency) were recorded. As a part of the dietary questionnaire, subjects reported their monthly intake of sheep meat (lamb and mutton), total meat intake (15 times/month, 15–25 times/month or more than 25 times/month), and fish intake (zero fish intake, 1–10 times per month or more than 10 times per month). According to monthly sheep meat intake frequency (SMIF groups), the subjects were classified into three groups for further analysis, which are zero SMIF group (less than one time per month), moderate SMIF group (1–10 times per month) and high SMIF group (more than 10 times per month).

### Chemicals and reagents

Chemicals and reagents used in this study were purchased from Sigma-Aldrich (Søborg, Denmark). These included deuterium oxide (D_2_O, 99.9 atom % D), monobasic sodium phosphate (NaH_2_PO_4_, ≥ 99.0%), and dibasic sodium phosphate (Na_2_HPO_4_, ≥ 98.0%), sodium salt of 3-(trimethylsilyl) propionic-2,2,3,3-d4 acid (TSP, 98 atom % D, ≥ 98.0%), and sodium azide (NaN_3_, ≥ 99.5%). The water used throughout the study was purified using a Millipore lab water system (Merck KGaA, Darmstadt, Germany) equipped with a 0.22 μm filter membrane.

### Sample collection and preparation for ^1^H NMR analysis

Fasting blood plasma samples were collected into EDTA-containing tubes and subsequently stored in cryovials at − 80 °C until ^1^H NMR measurements. Prior to preparation, plasma samples were thawed at room temperature for approximately 30 min. Aliquots of 350 μL of plasma were gently mixed with equal amounts of phosphate buffer in 2.0 mL Eppendorf tubes. The phosphate buffer, based on NaH_2_PO_4_ and Na_2_HPO_4_ solutions, was prepared as previously described (Beckonert et al., 2007). 600 µL of plasma buffer mixture was then transferred into 5 mm O.D. (103.5 mm length) SampleJet tubes (Bruker BioSpin, GmbH, Germany). Sample preparation and measurements were randomized. Pooled control human blood plasma samples were measured at regular intervals throughout the whole measurement sequence.

### ^1^H NMR spectral data acquisition and processing

The ^1^H NMR spectra of blood plasma samples were acquired as previously described (Khakimov et al., 2022). Briefly, spectra were acquired using a Bruker Avance III 600 MHz NMR spectrometer equipped with a 5-mm broadband inverse (BBI) probe, automated tuning and matching accessory (ATMA) and a cooling unit (BCU-05). The spectrometer was equipped with an automated sample changer (SampleJet, Bruker BioSpin, Rheinstetten, Germany) hosting with a refrigerated sample storage station with cooling racks (278 K) and a heating/drying station (298 K). Data acquisition and processing were carried out using TOPSPIN 3.5 (Bruker BioSpin, Rheinstetten, Germany) and automation of the overall measurement procedure was controlled by IconNMR™ (Bruker BioSpin, Rheinstetten, Germany). Prior to NMR measurements, each sample was pre-heated at 306 K for 60 s in the SampleJet and was kept inside the probe head for 5 min to reach temperature equilibrium at 310 ± 0.1 K. Automated tuning and matching, automated locking, and automated shimming (TOPSHIM routine) were performed for each sample. Automation included also the 90° hard pulse calibration, and optimised presaturation power for each sample. The ^1^H NMR spectra were acquired using the pulse sequence for water suppression 1D NOESY (*noesygppr1d*, Bruker nomenclature) and 32 scans, which were collected into 131,072 data points using a spectral width of 30 ppm, a 90° pulse, a recycle delay (d1) of 4 s and a mixing time of 0.01 s. The receiver gain value was kept constant for all samples (90.5). A second series of ^1^H NMR spectra was recorded using the CPMG-presat pulse sequence (*cpmgpr*, Bruker nomenclature). A total of 32 scans were acquired after 4 dummy scans, and the generated free induction decays (FIDs) were collected into 96 k data points using a spectral width of 30 ppm. The relaxation delay and mixing time were set to 4.0 and 0.01 s, respectively. The receiver gain was set to 90.5 for all samples. For each ^1^H NMR spectrum, automated data processing, including Fourier transform, apodization (LB = 0.3 Hz), automated phasing, and baseline correction was carried out in the TOPSPIN software. All NMR spectra were imported into the SigMa software (Khakimov et al., 2020). The spectra were then scaled in the case of NOESY to the ERETIC (Electronic REference To access In vivo Concentrations) signal (Akoka et al., 1999), positioned at 15 ppm, which is equivalent to 10 mmol/L protons, and aligned towards the doublet of alanine’s methyl group (1.507 − 1.494 ppm) using *icoshift* (Savorani et al., 2010).

### Statistical data analysis

**Fig. 1:**
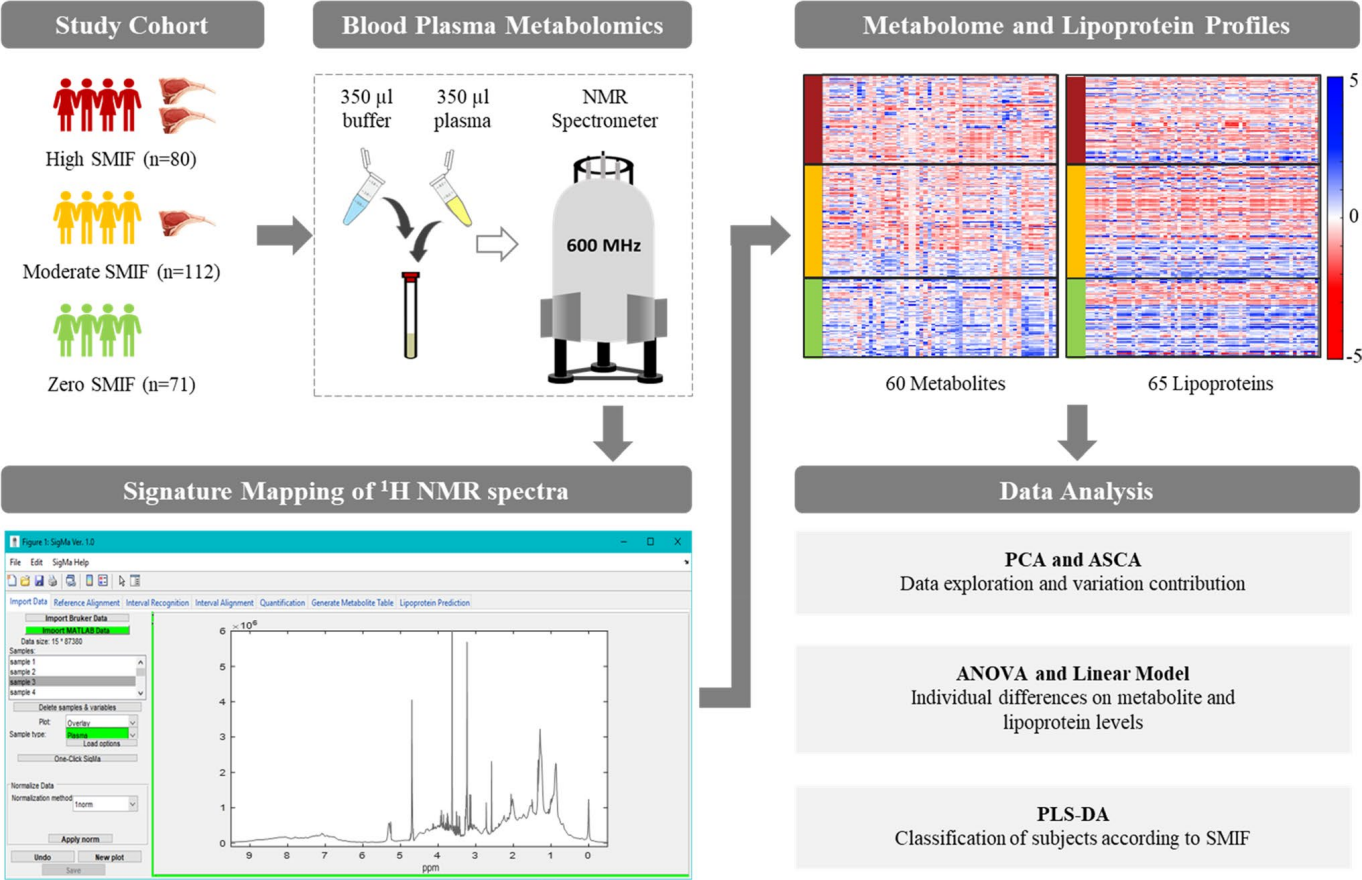
Study Design and workflow. A total of 263 healthy Uzbek adults were recruited, which were divided into three groups based on their sheep meat intake frequency (SMIF): high SMIF (> 10 times per month), moderate (1–10 times per month) and zero (reporting less than 1 time per month) SMIF. Fasting plasma of all individuals were collected and subjected to proton nuclear magnetic resonance (^1^H NMR)-based metabolomics. Blood plasma metabolome profile (60 metabolites) and 65 lipoproteins were quantified from ^1^H NMR spectra by SigMa software. To reveal the possible effects of SMIF, statistical analyses methods including principal component analysis (PCA), ANOVA-simultaneous component analysis analysis (ASCA), one-way analysis of variance (ANOVA), linear model and partial least squares discriminant analysis (PLS-DA) were applied on the metabolome and lipoprotein datasets separately

Differences in blood plasma metabolites and lipoproteins between the three SMIF groups were evaluated using one-way analysis of variance (ANOVA) with Benjamini–Hochberg’s false discovery rate (FDR-p) correction (10%). FDR-p value < 0.05 was considered as the significant threshold. Multiple linear regression (MLR) analysis was used to test the effect of SMIF on metabolites or lipoproteins. Separate model was developed for each metabolite or lipoprotein variable, with metabolites or lipoproteins as dependent variable and SMIF groups as independent variable (as categorical variable, class 1 = zero, 2 = moderate, 3 = high) adjusted for nationality (categorical variable, 1 = Uzbek, 2 = non-Uzbek), sex (categorical variable, 1 = males, 2 = females), BMI (continuous variable), age (continuous variable), total meat intake (as categorical variable, 1 = less than 15 times/month, 2 = between 15–25 times/month, 3 = more than 25 times/month) and fish intake frequency (as categorical variable, 1 = less than once a month, 2 = between 1–10 times/month, 3 = more than 10 times/month). Multivariate analyses, including principal component analysis (PCA) (Hotelling, 1933), ANOVA-simultaneous component analysis (ASCA) (Smilde et al., 2005), and partial least squares-discriminant analysis (PLS-DA) (Ståhle & Wold, 1987) were performed in order to explore the data and identify the metabolites discriminating the three SMIF groups. The procedure used for PLS-DA model optimization and validation have been previously described elsewhere (Khakimov et al., 2016). The study design is schematically represented in Fig. 1. All statistical analyses were performed using MATLAB (version R2016ba, the Mathworks, Inc., Natick, MA, USA) and customised MATLAB scripts.

## Results

### Characteristics of the study population

A total of 263 healthy adults, 149 females and 114 males, between the age of 18 and 85 years (46.94 ± 15.91) were included in the study. The subjects were stratified into three groups based on the SMIF: (1) subjects reported to consume sheep meat > 10 times per month (high SMIF, n = 80), (2) sheep meat consumption equals to1-10 times per month (moderate SMIF, n = 112) and (3) less than one time per month (zero SMIF, n = 71). Table 1 lists the main characteristics of the study subjects. Nationality, sex, BMI, and age were partially confounded with the SMIF as found from the chi-square analysis (*p*-value < 0.05). The intake frequency of total meat and fish were also significantly different in three SMIF groups (p-value < 0.05). Subsequently these confounding factors were adjusted for in a following multiple linear regression model when evaluating the SMIF effect (Table 2). In general, females, older subjects, and subjects with BMI > 27 kg/m^2^ have less than one time per month sheep meat intake (zero SMIF group). The effect of sex, BMI and age on blood plasma metabolites and lipoproteins will be described in a separate article. Smoking, alcohol consumption and blood glucose levels were found not to be different between the three SMIF groups. The average level of blood cholesterol was 3.71 ± 0.68 mmol/L in the high SMIF group, 3.73 ± 0.60 mmol/L in the moderate SMIF group and 4.05 ± 0.84 mmol/L in the zero SMIF group (*p*-value = 0.004).

**Table 1:** Characteristics of the study subjects

	All	Sheep meat intake frequency (times per month)			
	(n = 263)	> 10 (high, n = 80)	1–10 (moderate, n = 112)	0 (zero, n = 71)	*p-*value
Sex					0.011
Female	149 (56.7%)	37 (46.3%)	62 (55.4%)	50 (70.4%)	
Male	114 (43.3%)	43 (53.8%)	50 (44.6%)	21 (29.6%)	
Nationality					< 0.001
Uzbek	229 (87.1%)	77 (96.3%)	101 (90.2%)	51 (71.8%)	
Other	34 (12.9%)	3 (%)	11 (9.8%)	20 (28.2%)	
Age, years	46.94 ± 15.91	43.99 ± 14.10	43.71 ± 16.30	55.43 ± 14.22	< 0.001
BMI, kg/m^2^	25.83 ± 4.79	26.04 ± 4.54	24.82 ± 4.82	27.21 ± 4.74	0.005
Smoking	49 (18.6%)	14 (17.5%)	25 (22.3%)	10 (14.1%)	0.360
Total meat (times per month)					< 0.001
> 25	173	56 (70.0%)	76 (67.9%)	41 (57.7%)	
15–25	52	21 (26.2%)	23 (20.5%)	8 (11.3%)	
< 15	38	3 (3.8%)	13 (11.6%)	22 (31.0%)	
Fish (times per month)					0.002
> 10	7 (2.7%)	1 (1.2%)	6 (5.4%)	0	
1–10	203 (77.2%)	68 (85.0%)	88 (78.6%)	47 (66.2%)	
0 (zero)	53 (20.1%)	11 (13.8%)	18 (16.0%)	24 (33.8%)	
Alcohol consumption (times per month)					0.724
> 5	8 (3.0%)	4 (5.0%)	3 (2.7%)	1 (1.4%)	
1–5	61 (23.2%)	19 (23.8%)	27 (24.1%)	15 (21.1%)	
0	194 (73.8%)	57 (71.2%)	82 (73.2%)	55 (77.5%)	
Blood glucose (mmol/L)	4.35 ± 0.77	4.31 ± 0.78	4.40 ± 0.79	4.31 ± 0.71	0.661
Blood cholesterol (mmol/L)	3.81 ± 0.71	3.71 ± 0.68	3.73 ± 0.60	4.05 ± 0.84	0.004

Data are given as mean ± SD, or n (%). *p-*values were calculated from the 1-factor ANOVA for continuous variables and χ^2^ test for categorical variables. *Data come from a self-reported questionnaire

**Table 2:** List of blood plasma metabolites and lipoproteins found to be different between the three sheep meat intake frequency (SMIF) groups by ANOVA and multiple linear modelling

	Fold change^b^		*p-value*^c^	Effect (%)	*p-value*^d^						
	H/Z^a^	M/Z^a^			SMIF	Age	Sex	BMI	Meat	Fish	Nationality
Metabolites											
Pyruvic acid	0.61	0.81	< 0.001	12.62	< 0.001	< 0.001	0.039	0.038	0.612	0.996	0.976
Phenylalanine	0.76	0.85	< 0.001	9.04	0.009	< 0.001	0.043	0.907	0.888	0.524	0.290
Ornithine	0.68	0.88	< 0.001	8.06	< 0.001	0.002	< 0.001	0.612	0.612	0.996	0.560
Acetic acid	0.69	0.83	0.002	5.92	0.009	< 0.001	0.007	0.241	0.888	0.346	0.988
Formate	0.66	0.8	0.007	4.90	0.032	< 0.001	0.103	0.759	0.934	0.276	0.360
Choline	1.11	1.06	< 0.001	8.23	0.002	0.002	0.748	0.449	0.888	0.539	0.742
Bin (DMG, Asn)	1.33	1.19	< 0.001	9.91	0.004	< 0.001	0.399	0.933	0.888	0.996	0.988
Bin (Asn)	1.08	1.02	0.001	7.30	0.002	0.367	0.597	0.104	0.612	0.524	0.484
Bin (serine)	1.09	1.04	0.002	6.28	0.005	0.108	0.554	0.001	0.612	0.972	0.271
Bin (Glu, Tym, His, TMAO, Bet, Arg, Tau)	0.92	0.95	0.003	5.81	0.024	< 0.001	0.001	0.166	0.913	0.996	0.595
Bin (3-hydroxybutyric acid)	1.10	1.02	0.015	4.30	0.076	0.268	0.425	0.196	0.612	0.996	0.350
bin (proline)	0.89	0.91	0.038	3.24	0.024	< 0.001	< 0.001	0.155	0.888	0.346	0.289
Lipoproteins											
Plasma *chol*	0.81	0.88	< 0.001	7.53	0.135	< 0.001	0.012	0.075	0.929	0.995	0.066
Plasma *chole*	0.81	0.87	< 0.001	7.41	0.210	< 0.001	0.908	0.085	0.929	0.995	0.066
Plasma *apoA1*	0.87	0.95	0.001	6.06	0.154	< 0.001	0.056	0.063	0.929	0.995	0.066
LDL-4-*chol*	0.76	0.83	< 0.001	7.88	0.139	< 0.001	0.572	0.974	0.958	0.995	0.120
LDL-3-*chole*	0.74	0.82	< 0.001	7.34	0.138	< 0.001	0.348	0.771	0.929	0.995	0.066
HDL-*chol*	0.77	0.93	< 0.001	6.79	0.210	< 0.001	0.702	0.096	0.929	0.995	0.066
HDL-2b-*chol*	0.75	0.94	0.003	5.09	0.161	< 0.001	0.538	0.119	0.929	0.995	0.066
HDL-2b-*chole*	0.67	0.87	0.001	6.69	0.249	0.232	0.002	0.103	0.929	0.995	0.263
HDL-*apoA1*	0.82	0.92	< 0.001	8.40	0.210	< 0.001	0.695	0.096	0.929	0.995	0.066
HDL-2a-*apoA1*	0.82	0.92	< 0.001	6.34	0.139	0.030	0.043	0.111	0.970	0.995	0.178
HDL-3-*apoA1*	0.83	0.89	< 0.001	6.89	0.139	< 0.001	0.452	0.040	0.929	0.995	0.066

*DMG* dimethylglycine, *Asn* asparagine, *Glu* glucose, *Tym* tyramine, *His* histidine, *TMAO* Trimethylamine N-oxide, *Bet* betaine, *Arg* arginine, *Tau* taurine

^a^H = high frequency sheep meat intake (> 10 times per month. n = 80). M = moderate frequency sheep meat intake (1–10 times per month. n = 112). Z = zero sheep meat intake (less than one time per month. n = 72)

^b^Fold change was calculated on the mean abundances between the two SMIF groups (high by zero (H/Z) and moderate by zero (M/Z))

^c^*p*-values of ANOVA between the three SMIF groups are corrected for the false discovery rate (FDR, 5%)

^d^*p*-values from the multiple linear modelling (FDR, 5%)

### Effect of sheep meat intake on blood plasma metabolites

**Fig. 2:**
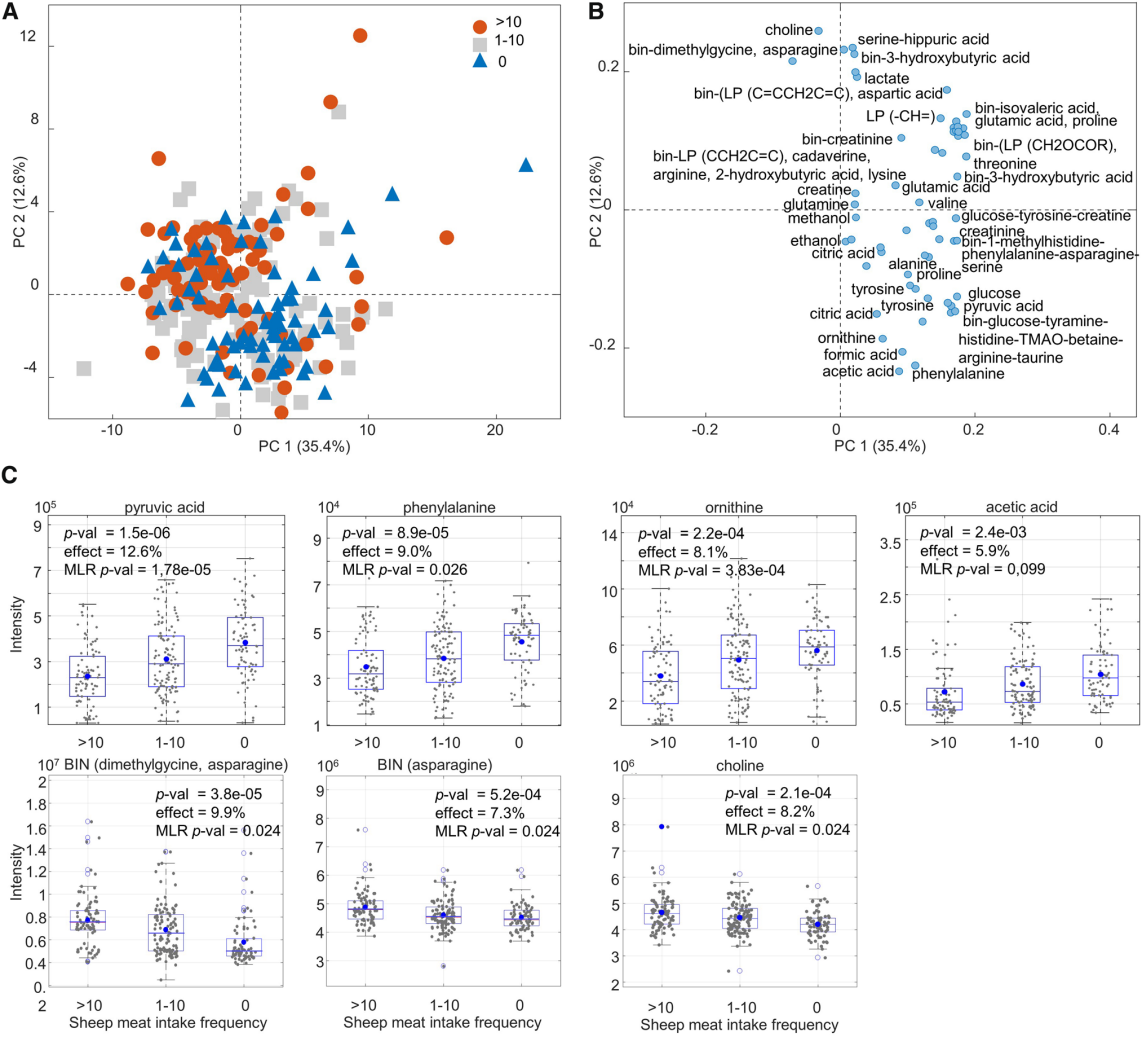
Blood plasma metabolites found to be associated to the sheep meat intake frequency (SMIF) in healthy Uzbek adults. **A** Scores and **B** loadings plots of the principal component analysis (PCA) model developed on blood plasma metabolomics data. **C** Box plots of the discriminant metabolites pyruvic acid, phenylalanine, ornithine, acetic acid, dimethylglycine, asparagine, and choline. The horizontal lines and dots inside the boxes represent median and mean values, respectively. *p*-values and effect sizes (%) are calculated using ANOVA. Multiple linear regression model (MLR) *p*-values represents false discovery rate (FDR) corrected *p*-values from multiple linear regression analysis adjusted for sex, age and BMI

**Fig. 3:**
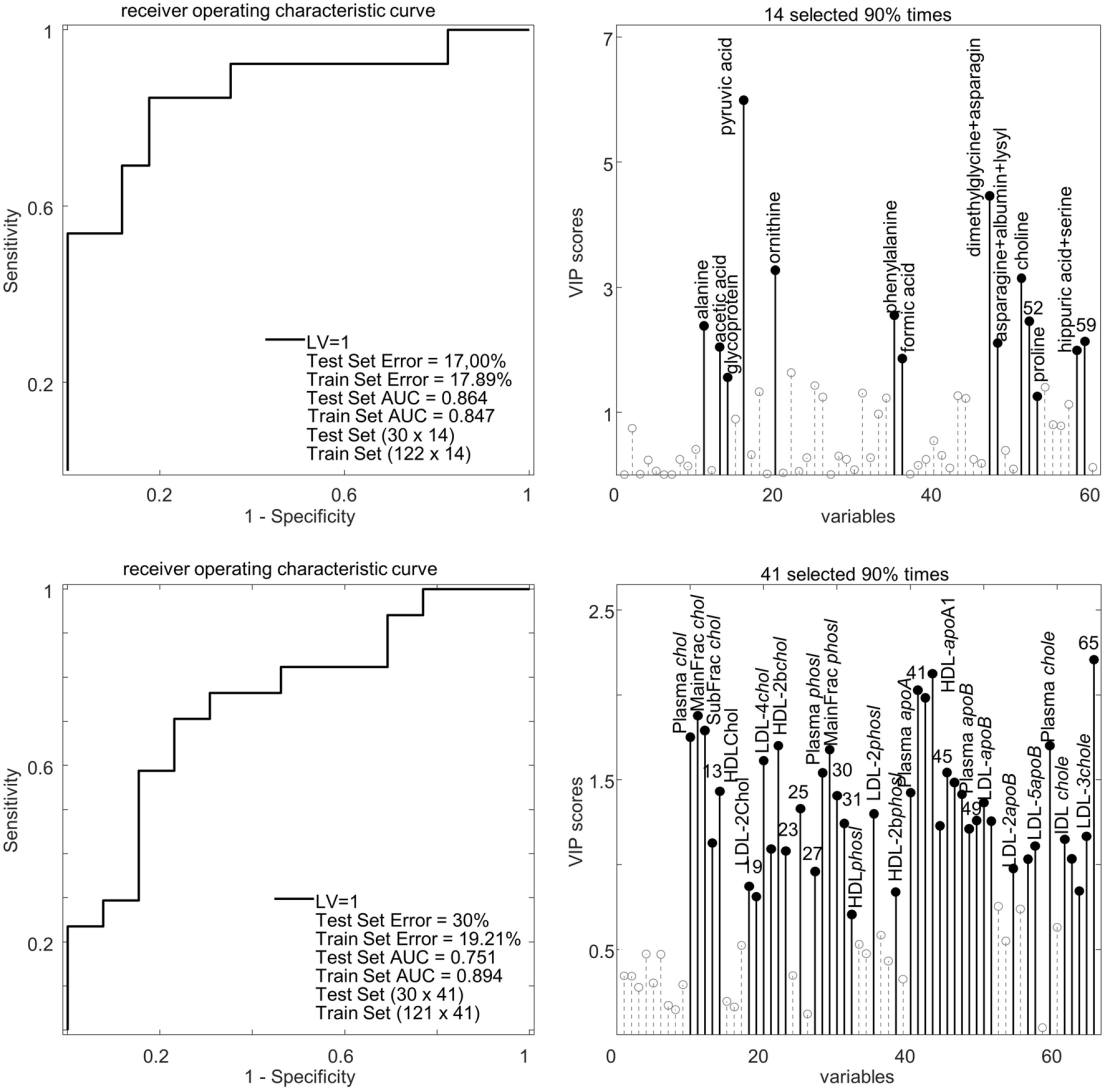
Classification performances of partial least squares-discriminant analysis (PLS-DA) models developed to classify high and zero sheep meat intake frequency (SMIF, more than 10 times/month, 1–10 times/month, less than 1 time/month) subjects using the NMR based blood plasma metabolomics data (top panel) and lipoprotein data (bottom panel). Left graphs show the area under the curve (AUC) of receiver operating characteristic (ROC) curve of the PLS-DA models. AUC and ERROR (misclassification rate) are shown both for the test set prediction, using independent subjects, and training models. The graphs to the right show selectivity ratios of metabolites and lipoproteins identified to be strong discriminants for classifying the high SMIF and zero SMIF subjects using a PLS-DA variable selection approach. The metabolite and lipoprotein variables showed in the plots were listed in Table S1 and S2, respectively. Both PLS-DA models show a moderate classification power with misclassification rates of 17 and 30% and AUC of 0.75–0.89 for training and test set models, respectively. *LV latent variables, *VIP* variable importance projection, *LDL* low-density lipoprotein, *Chol* cholesterol, *HDL* high-density lipoprotein, *Phosl* phospholipid, *Mainfrac* main fraction, *Subfrac* subfraction, *ApoA1* apolipoprotein A1, *ApoB* apolipoprotein B, *chole* cholesterol ester

A total of 60 blood plasma metabolites, corresponding to 36 signature signals (SS), and 24 signals of complex intervals representing many overlapped and/or shifted signals (BINS) were detected from the CMPG spectra using SigMa (Table S1). Relative concentrations of SS were determined using the multivariate curve resolution algorithm (Lawton & Sylvestre, 1971) implemented in SigMa. The metabolomics data was then subjected to univariate and multivariate data analyses to evaluate a possible effect of SMIF. Principal component analysis (PCA) performed on the metabolomics data revealed a partial separation of high *versus* zero SMIF groups (Fig. 2A). The moderate SMIF group overlapped with the two other groups. Based on the loadings of the corresponding PCA model, the separation between high and zero SMIF groups is mainly attributed to relatively higher levels of pyruvic acid, phenylalanine, ornithine, formic acid, acetic acid, proline, and alanine in the zero SMIF group, while choline, serine, and complex spectral intervals dominated by signature signals of dimethylglycine and asparagine were found to be higher in the high SMIF group (Fig. 2B). The SMIF-related variability distribution for the above-mentioned metabolites is shown in Fig. 2C as box and whiskers plots (*p*-value < 0.05). The same metabolites were found to be the strongest discriminating variables for classifying high SMIF subjects from zero SMIF subjects using a PLS-DA modelling (Fig. 3). The optimised PLS-DA model after variable selection showed a reasonable classification power (error = 17.0%, AUC = 0.86) on an independent test set (40% subjects). ASCA revealed that the SMIF effect was significant (*p* = 0.0001), explaining 2.8% of the total variation in the metabolomics dataset. In addition, the effect was evaluated using One-Way ANOVA on the individual metabolite levels which allowed to identify 23 blood plasma metabolites being different between the three SMIF groups after FDR correction (Table S1).

The confounding factors nationality, sex, BMI, age, intake frequency of total meat and fish identified to be significant from the chi-square test (Table 1), were further evaluated for their possible interference with the SMIF effect. A MLR model was used for this purpose where the confounding factors were treated as fixed effects while testing the SMIF effect (see materials and methods). Twelve metabolites remained significant for the SMIF effect after adjusting for the six confounding factors, which were also found to be related to the SMIF in PCA and PLS-DA analyses (Table 2). Among them, the blood plasma concentrations of pyruvic acid, phenylalanine, ornithine, acetic acid, formate and proline were found at higher levels in zero and moderate SMIF groups compared to the high SMIF group. In contrast, the blood plasma levels of dimethylglycine, asparagine, choline, serine and 3-hydroxybutyric acid were found to be weakly positively correlated to the SMIF. Further, the SMIF effect was evaluated after stratifying the subjects by sex and largely all metabolites that were found to be associated with the SMIF in the global ANOVA (including both males and females) remained significant for both sexes. However, MLR analysis after treating age, BMI, nationality and intake frequency of total meat and fish as fixed effects revealed that only the most affected metabolites in the global ANOVA analysis remained significant when stratified by sex (Table S1). This included pyruvic acid and ornithine in the MLR model of female and male subgroups and both metabolites decreased as SMIF increased. For males, also dimethylglycine, asparagine, and choline remained significant in MLR analysis and increased as SMIF increased. Likewise, SMIF, plasma metabolomics data was also scrutinized in terms of total meat intake frequency and fish intake frequency. The result showed that there were no differences on individual metabolites or LP variables from ANOVA after FDR correction (data not shown). Only creatine was found to be significantly different between subjects with different fish intake frequency in linear model (Table S1).

### Effect of sheep meat intake on plasma lipoproteins

**Fig. 4:**
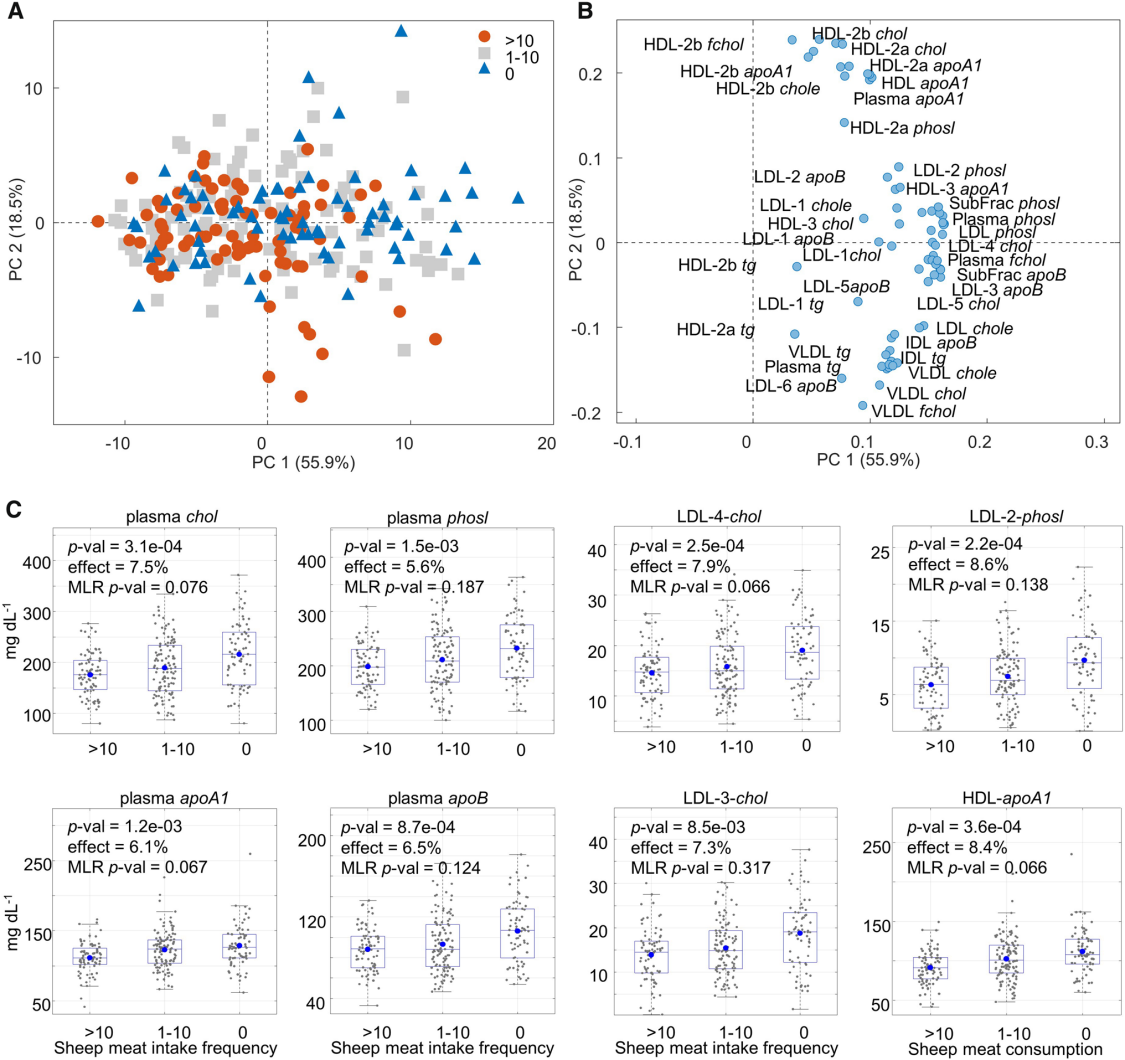
Human blood plasma lipoproteins associated with SMIF. (A) Scores and (B) loadings plot of the PCA model developed on the autoscaled concentrations of 65 plasma lipoprotein variables quantified in the three groups: high (more than 10 times/month, n = 80), moderate (1–10 times/month, n = 112) and zero (less than 1 time/month, n = 71) SMIF. (C) Box plots of selected plasma lipoproteins that are found to be different in the three SMIF groups. FDR corrected *p*-values and effect sizes (%) are calculated from ANOVA. The horizontal lines and dots inside the boxes represent median and mean values, respectively. Multiple linear regression (MLR) *p*-value represents false discovery rate (FDR) corrected p-value from multiple linear regression analysis adjusted by sex, BMI, and age

Sheep meat intake frequency was further investigated in relation to 65 human blood plasma lipoproteins (LP) quantified from the ^1^H NMR spectra as previously described (Khakimov et al., 2022). In contrast to the metabolomics data, PCA performed on the LP data showed no systematic variation related to the SMIF (Fig. 4), though ASCA depicted an effect of SMIF (4.5% variation, *p*-value < 0.0002). PLS-DA based variable selection identified 45 LP as discriminants for classifying the high SMIF subjects from zero SMIF subjects. These included cholesterol (*chol*), cholesterol ester (*chole*), free cholesterol (*fchol*), phospholipids (*phosl*), apolipoprotein A1 (*apoA1*), and apolipoprotein B (*apoB*) in plasma, total LDL and HDL as well as their subfractions (Table 2 and Fig. 3). All the 45 LP markers selected by the PLS-DA were also found to be affected by SMIF in One-Way ANOVA with effect sizes of 2.63–8.5% (Table S2) and they all displayed lower blood plasma levels in the high SMIF group compared to zero SMIF group (Fig. 4C). Fourteen of these LP variables including total blood plasma levels of *chol, chole,* and *apoA1,* and their levels in LDL and HDL subfractions remained significantly lower after adjusting for sex, BMI, age, nationality, intake frequency of total meat and fish in MLR analysis. The above-mentioned LP variables were 5–18% lower in the moderate SMIF group and 13–33% lower in the high SMIF group compared to the zero SMIF group. These LP variables were also consistently selected by the PLS-DA models developed to classify the high SMIF group in contrast to the zero SMIF group. However, none of these lipoproteins remained significant after FDR correction (Table 2 and Table S2). The LP data was subsequently evaluated for the effect of SMIF, separately for males and females. The effect of SMIF on the majority of LP variables decreased when studying males and females separately using MLR analysis. However, the MLR analysis after treating age, BMI, nationality, intake frequency of total meat and fish as fixed effects left no significant LP variables. Like metabolomics data, the LP data was also scrutinized in terms of total meat intake frequency and fish intake frequency. The result showed that there were no differences on individual LP variables from ANOVA after FDR correction (data not shown) neither from multivariate data analysis (Table S2).

## Discussion

Even though extensive metabolomics research has been conducted to better understand how red or white meat consumption impacts human metabolism, mostly using LC–MS based blood plasma or urine metabolomics (Goethals et al., 2020; Guan et al., 2021), the impact of sheep meat intake frequency on blood plasma metabolites or lipoproteins has not been studied yet. Most studies observed that red meat intake is associated with fatty acid and energy metabolism, and some found acylcarnitines being associated with red meat intake (Guan et al., 2021; Wedekind et al., 2020), but not to a specific type of red meat. In this study, we assessed the associations between sheep meat intake frequency and plasma metabolites and lipoprotein levels in a population screening study performed in Uzbekistan. Both univariate and multivariate statistical analyses, either performed on all subjects or stratified by sex, converged to a metabolic pattern consisting of a few metabolites and lipoproteins that were found to be consistently associated with the SMIF. The plasma metabolites associated with the SMIF included lower pyruvate, phenylalanine and acetate, and higher choline, dimethylglycine, and asparagine. These metabolites are mainly involved in protein biosynthesis, energy metabolism, and lipid metabolism in humans.

Based on the food consumption study conducted in Uzbekistan between 1992–2019, the food ingredient pattern of the Uzbek population is composed mainly of crops (wheat and rice) with meat, mainly beef and sheep meat (Mengmeng et al., 2022). People who have the highest meat intake in other countries have a lower consumption of vegetables and whole grains (Cosgrove et al., 2005). In this study, the high SMIF group not only had high intake frequency of sheep meat but also higher consumption of total meat intake frequency. Therefore, we hypothesise that the high SMIF group in this study had higher protein and fat intake in general and relatively low carbohydrate intake from grains and vegetables. The moderate SMIF and zero SMIF subjects are probably mixed with some subjects having high intakes of beef or milk or having an opposite dietary intake pattern with more wheat, oilseeds and fruits (Mengmeng et al., 2022). We observed that the SMIF has the strongest association with the blood plasma levels of pyruvate, which is a main intermediate product in the energy metabolism pathway. The pyruvate level was found to be 20–40% less in subjects who consumed sheep meat more than 10 times per month (high SMIF) compared to subjects who reported sheep meat intake less than once per month (zero SMIF groups). The ratio of pyruvate and lactate provides an estimate of the overall redox and energy state balance in the body because the NAD + /NADH ratio in cells is directly proportional to pyruvate/lactate ratio (Wijngaard et al., 2021). However, there was no difference of the plasma level of lactate between the three SMIF groups. The diet difference between the SMIF groups would result in low pyruvate without changing lactate and pressing the NAD + /NADH balance towards NAD + because NADH (and pyruvate) is used for gluconeogenesis, which is a main pathway where glucose is synthesized from certain non-carbohydrate carbon substrates. High fat and high protein diet increases the expression of phosphoenolpyruvate carboxykinase and glucose-6-phosphatase, which are two important enzymes at the start stage of gluconeogenesis (Moon & Koh, 2020; Rauckhorst et al., 2017) and lead to an increased usage of pyruvate. The metabolic markers for SMIF found in this study support the biological plausibility of our hypothesis. Further longitudinal studies are needed to prove a causal link between sheep meat intake and the change in blood plasma pyruvate levels.

Interestingly, the level of acetate, a main product of gut microbiota fermentation of dietary fibers in the colon, was found to be lower in high SMIF subjects, which might be explained by a lower intake of fibers in this group. Acetic acid is found in all living organisms and plays a vital role in carbohydrate and fat metabolism. Because of its importance in metabolism, the source of acetate is of considerable interest. Generally, exogenous acetate is the main source of acetate in vivo compared to dietary sources or endogenous acetate (Bose et al., 2019). The primary source of exogenous acetate is saccharolytic fermentation performed by the intestinal microbiota (Rey et al., 2010). Other food related sources of acetate are common in some foods such as process meat and cheese (Schug et al., 2016). However, these dietary sources, which are independent of the acetate formed by the gut microbiota, have relatively small contribution to the plasma levels of acetate. A small amount of acetate can also be generated endogenously by the deacetylation of proteins such as histones (Ye & Tu, 2018). In most studies, the concentration of SCFAs in feces, including acetic acid, correlates with a diet rich in dietary fibers (den Besten et al., 2013). We believe that the plasma concentration of acetate is positively related to higher consumption of fibres and that the high SMIF subjects consume less fibres than the zero SMIF subjects. Plasma formate also had this pattern in the initial analyses and formate levels in urine have previously been shown as a biomarker of fibre intake (Rasmussen et al., 2012). Another metabolite that had obviously different levels between the SMIF groups is ornithine. The high SMIF subjects showed 23 to 32% lower levels of blood plasma ornithine compared to moderate or zero SMIF subjects, respectively. Ornithine is a non-proteinogenic amino acid synthesized from arginine in the liver and thus enabling disposal of the excess nitrogen in the form of urea from the body. It is a central metabolite in the urea cycle and its blood plasma level in fasted subjects reflects the nitrogen metabolism in the body as a whole. Modelling studies indicate that plasma ornithine easily ends up in a stronger negative balance than most other amino acids under conditions of increased protein turnover (Dunstan et al., 2019). This may indicate that the high SMIF diets also increased the catabolism of this amino acid. A dietary intervention study showed that blood plasma levels of ornithine, proline and arginine were higher in subjects consuming grain-based proteins compared to subjects who consumed dairy or meat protein based diets (Altorf-van der Kuil et al., 2013). The inverse relation between plasma ornithine levels and SMIF found in our study may also be due to different sources of vegetable-based proteins in the diet of the three SMIF groups compared. This assumption needs further investigation of the Uzbek dietary pattern for validation.

Plasma levels of choline were 3 and 6% higher in high SMIF group compared to moderate or zero SMIF groups, respectively. Choline is an essential nutrient and plays a key role in multiple biosynthetic pathways as a source of methyl moiety, and it is a crucial component of cell membrane phospholipids. The main source of choline is food of animal origin, including milk, eggs, animal liver and meat. Several studies have shown that plasma levels of choline depend on the intake of foods rich in this metabolite, including red meat (Cho et al., 2016). Dimethylglycine is a metabolite of choline and therefore follows the same pattern.

Thirteen out of fourteen MLR significant lipoproteins were found at lower levels in the high SMIF class compared to zero SIMF, most of which are total plasma concentrations of *chol*, *chole*, and apoA1 as well as these molecules in LDL and HDL main and their subfractions. However, their difference among SMIF groups became insignificant after adjusting for confounders. These results suggest that the consumption of sheep meat may have less prominent effect on the plasma LP profile. Besides, no effect was observed in relation to total meat or fish intake on plasma LP profile. Instead, the difference of lipoproteins among SMIF groups might be due to the confounding factors such as age, sex, nationality and BMI. For example, the association between SMIF and plasma lipoproteins was decreased when studying females and males separately. Although the association between SMIF and plasma LP profile was not observed in our study, there are other studies which showed correlations between sheep meat intake and blood plasma level of cholesterol or fatty acids. A previous cross-over study of lamb meat intake has shown that reduction of blood plasma cholesterol might be due to a decrease in intestinal cholesterol absorption (Baila-Rueda et al., 2015). Some studies have reported that stearic acid (C18:0), which is known to reduce blood plasma LDL cholesterol level, is the most prominent saturated fatty acid in sheep meat (Li et al., 2005; Wang et al., 2021). These previous studies support the fact that the negative association between cholesterol in different lipoprotein particles and SMIF might be partially due to the high level of stearic acid in sheep meat. Sheep meat intake frequency may therefore have an impact on lowering cholesterol level in plasma. Relatively small sample size and multiple confounding factors considered in this study are probably the two major reasons why the changes in plasma LP profiles could not be linked with the SMIF. However, the results presented in our study warrant more structured and rigorous analyses in future investigation on sheep meat intake frequency and lipoprotein particles which correlate with the risk of atherosclerosis and CVD.

To the best of authors’ knowledge, this is the first attempt to study associations between human blood plasma metabolites/lipoproteins and sheep meat intake frequency. The present findings point out the importance of metabolomics and lipoprotein research for better understanding metabolic changes related to sheep meat intake frequency and the identification of robust dietary biomarkers specific for sheep meat intake. However, there are some limitations in this study. Firstly, the information on physical activity and the intake of other food items were not collected in a systematic way which hampered us to include these factors when evaluating the effect of SMIF on the plasma metabolome or lipoprotein profiles. Besides, the food consumption was not quantified, hence the consumed calories couldn’t be calculated and considered in the study. Further comparative research is required to evaluate our findings, especially in relation to a population that has a different red meat intake and with better information about the dietary macronutrients and dietary fiber intake. As an observational study, it was not possible to determine a causal relationship between blood plasma levels of metabolites or lipoproteins and the consumption of sheep meat, thus further investigations are needed to better understand changes in human metabolism and possible alterations in the biochemical pathways regulating digestion of animal protein and fat after sheep meat intake.

## Conclusion

The study demonstrates that, regardless of confounding factors (sex, BMI, age, nationality, total meat or fish intake frequency), some plasma metabolites showed significant and consistent trends associated with the SMIF. This metabolic pattern explaining SMIF included plasma metabolites such as pyruvate, phenylalanine, ornithine, formate and acetic acid, which were lower in the high SMIF group, and choline, asparagine, and dimethylglycine, that were higher in the high SMIF group; both as compared to the zero SMIF group. In addition, several lipoproteins including, levels of *chol, chole,* and *apoA1* in plasma, and total LDL and HDL and their subfractions were found to be lower in the high SMIF group compared to zero SMIF group although the difference were not significant after FDR correction.

## Supplementary Information

Below is the link to the electronic supplementary material.

Supplementary file1 (DOCX 50 KB)

Supplementary file2 (DOCX 46 KB)

## Data Availability

The datasets generated during and/or analyzed during the current study are available from the corresponding author on reasonable request.
