# Supplementary material for: Associations between sheep meat intake frequency and blood plasma levels of metabolites and lipoproteins in healthy Uzbek adults
Source: Metabolomics. 2023 Apr 26;19(5):46. doi: 10.1007/s11306-023-02005-x (PMC10133350; doi:10.1007/s11306-023-02005-x)
Supplement: Supplementary file 1 — Supplementary file1 (DOCX 50 KB) [file 11306_2023_2005_MOESM1_ESM.docx]

Table S1. Human blood plasma metabolite table generated from ^1^H NMR Carr–Purcell–Meiboom–Gill (CPMG) spectra.

| N | metabolite name | Class^#^ | chemical shift (ppm) | multiplicity* | Loadings of PCA model | | SMIF** | | | | SMIF*** | Age*** | Sex*** | BMI*** | Total meat*** | Fish*** | Nationality  *** |
| --- | --- | --- | --- | --- | --- | --- | --- | --- | --- | --- | --- | --- | --- | --- | --- | --- | --- |
|  |  |  |  |  | PC 1 | PC 2 | FDR-p-value | FDR-p-value | FC H/Z | FC M/Z | FDR-p-value | FDR-p-value | FDR-p-value | FDR-p-value | FDR-p-value | FDR-p-value | FDR-p-value |
| 1 | cholesterol | SS | 0.71 | s | 0.15 | 0.13 | 0.13 | 2.16 | 1.14 | 0.96 | 0.609 | <0.001 | 0.008 | 0.068 | 0.888 | 0.996 | 0.503 |
| 2 | LP(C***H_3_***) | SS | 0.89 | m | 0.18 | 0.11 | 0.11 | 2.34 | 0.97 | 0.86 | 0.901 | <0.001 | 0.425 | 0.068 | 0.915 | 0.996 | 0.348 |
| 3 | isoleucine | SS | 0.97 | d (t) | 0.13 | -0.02 | 0.92 | 0.16 | 1.00 | 1.02 | 0.970 | 0.027 | <0.001 | 0.001 | 0.888 | 0.996 | 0.484 |
| 4 | leucine | SS | 0.99 | s (t) | 0.13 | -0.07 | 0.91 | 0.15 | 0.98 | 0.98 | 0.584 | 0.001 | <0.001 | 0.003 | 0.888 | 0.996 | 0.595 |
| 5 | valine | SS | 1.01 | d | 0.12 | 0.01 | 0.35 | 1.10 | 1.03 | 1.05 | 0.547 | 0.624 | <0.001 | <0.001 | 0.888 | 0.996 | 0.35 |
| 6 | isoleucine | SS | 1.03 | d | 0.14 | -0.02 | 0.95 | 0.08 | 1.01 | 1.02 | 0.970 | 0.069 | <0.001 | 0.001 | 0.888 | 0.996 | 0.424 |
| 7 | valine | SS | 1.07 | d | 0.14 | -0.02 | 0.78 | 0.30 | 1.02 | 1.02 | 0.844 | 0.038 | <0.001 | <0.001 | 0.747 | 0.996 | 0.306 |
| 8 | propylene glycol | SS | 1.14 | d | 0.06 | -0.06 | 0.51 | 0.66 | 0.97 | 0.96 | 0.460 | 0.947 | 0.001 | 0.442 | 0.888 | 0.996 | 0.271 |
| 9 | LP (C***H_2_***) | SS | 1.29 | m | 0.17 | 0.11 | 0.29 | 1.25 | 1.07 | 0.86 | 0.903 | <0.001 | 0.01 | 0.036 | 0.98 | 0.996 | 0.424 |
| 10 | lactate | SS | 1.35 | d | 0.03 | 0.19 | 0.37 | 1.01 | 1.06 | 0.97 | 0.900 | 0.239 | 0.201 | 0.023 | 0.888 | 0.601 | 0.945 |
| 11 | alanine | SS | 1.50 | d | 0.13 | -0.07 | 0.038 | 3.26 | 0.96 | 0.89 | 0.156 | 0.002 | 0.009 | 0.03 | 0.612 | 0.524 | 0.496 |
| 12 | LP (C***H_2_***CCO) | SS | 1.59 | m | 0.17 | 0.12 | 0.25 | 1.45 | 1.16 | 0.89 | 0.900 | <0.001 | 0.004 | 0.031 | 0.98 | 0.996 | 0.484 |
| 13 | acetic acid | SS | 1.94 | s | 0.09 | -0.23 | 0.0024 | 5.92 | 0.84 | 0.69 | 0.009 | <0.001 | 0.007 | 0.241 | 0.888 | 0.346 | 0.988 |
| 14 | glycoprotein (acetyls) | SS | 2.06 | s | 0.15 | 0.08 | 0.0058 | 5.13 | 0.99 | 0.91 | 0.569 | 0.002 | 0.183 | <0.001 | 0.934 | 0.996 | 0.327 |
| 15 | glutamic acid | SS | 2.37 | m | 0.08 | 0.04 | 0.17 | 1.88 | 0.97 | 0.88 | 0.549 | 0.186 | 0.008 | 0.047 | 0.888 | 0.996 | 0.496 |
| 16 | pyruvic acid **^f, m^** | SS | 2.39 | s | 0.11 | -0.11 | <0.001 | 12.62 | 0.76 | 0.61 | <0.001 | <0.001 | 0.039 | 0.038 | 0.612 | 0.996 | 0.976 |
| 17 | citric acid | SS | 2.55 | s (d) | 0.04 | -0.08 | 0.36 | 1.06 | 0.97 | 0.94 | 0.614 | <0.001 | <0.001 | 0.068 | 0.934 | 0.996 | 0.976 |
| 18 | citric acid | SS | 2.66 | s (d) | 0.06 | -0.15 | 0.025 | 3.83 | 0.94 | 0.90 | 0.900 | <0.001 | 0.008 | 0.064 | 0.98 | 0.996 | 0.909 |
| 19 | citric acid | SS | 2.69 | s (d) | 0.06 | -0.05 | 0.66 | 0.46 | 1.00 | 0.96 | 0.705 | <0.001 | 0.057 | 0.819 | 0.972 | 0.996 | 0.976 |
| 20 | ornithine **^f, m^** | SS | 3.08 | d (t) | 0.06 | -0.19 | <0.001 | 8.06 | 0.77 | 0.68 | <0.001 | 0.002 | <0.001 | 0.612 | 0.612 | 0.996 | 0.56 |
| 21 | methanol | SS | 3.38 | s | 0.02 | -0.01 | 0.99 | 0.03 | 1.00 | 1.01 | 0.900 | 0.468 | 0.001 | 0.607 | 0.888 | 0.524 | 0.271 |
| 22 | glucose | SS | 3.51 | m | 0.16 | -0.15 | 0.014 | 4.28 | 0.96 | 0.90 | 0.271 | <0.001 | 0.037 | 0.389 | 0.612 | 0.996 | 0.325 |
| 23 | ethanol | SS | 3.66 | s (q) | 0.01 | -0.05 | 0.98 | 0.03 | 1.10 | 1.00 | 0.927 | 0.939 | 0.743 | 0.933 | 0.888 | 0.996 | 0.976 |
| 24 | ethanol | SS | 3.68 | t (q) | 0.02 | -0.04 | 0.92 | 0.12 | 0.87 | 0.85 | 0.900 | 0.911 | 0.76 | 0.717 | 0.888 | 0.996 | 0.595 |
| 25 | glucose | SS | 3.86 | m | 0.17 | -0.14 | 0.027 | 3.73 | 0.96 | 0.91 | 0.362 | <0.001 | 0.088 | 0.402 | 0.612 | 0.996 | 0.271 |
| 26 | Creatine | SS | 3.95 | s | 0.02 | 0.02 | 0.032 | 3.48 | 0.77 | 0.81 | 0.415 | 0.095 | <0.001 | 0.058 | 0.888 | 0.011 | 0.976 |
| 27 | creatinine | SS | 4.07 | s | 0.10 | -0.03 | 0.99 | 0.01 | 1.00 | 1.00 | 0.900 | <0.001 | <0.001 | 0.717 | 0.888 | 0.996 | 0.271 |
| 28 | lactate | SS | 4.14 | q | 0.02 | 0.20 | 0.41 | 0.88 | 1.07 | 0.99 | 0.927 | 0.135 | 0.211 | 0.016 | 0.888 | 0.597 | 0.945 |
| 29 | proline | SS | 4.16 | s (dd) | 0.10 | -0.09 | 0.49 | 0.71 | 0.94 | 0.93 | 0.271 | <0.001 | <0.001 | 0.717 | 0.888 | 0.996 | 0.484 |
| 30 | LP (C***H***OCOR) | SS | 5.21 | m | 0.17 | 0.11 | 0.23 | 1.55 | 1.12 | 0.89 | 0.900 | <0.001 | 0.008 | 0.041 | 0.98 | 0.996 | 0.484 |
| 31 | glucose | SS | 5.25 | d | 0.16 | -0.13 | 0.038 | 3.20 | 0.96 | 0.92 | 0.419 | <0.001 | 0.085 | 0.339 | 0.612 | 0.996 | 0.306 |
| 32 | LP (-C***H***=) | SS | 5.32 | m | 0.17 | 0.13 | 0.19 | 1.71 | 1.03 | 0.87 | 0.960 | <0.001 | 0.102 | 0.031 | 0.934 | 0.996 | 0.36 |
| 33 | tyrosine | SS | 6.92 | m | 0.10 | -0.11 | 0.28 | 1.31 | 0.99 | 0.95 | 0.900 | 0.013 | 0.11 | <0.001 | 0.98 | 0.996 | 0.484 |
| 34 | tyrosine | SS | 7.21 | m | 0.13 | -0.13 | 0.084 | 2.58 | 1.00 | 0.92 | 0.800 | <0.001 | 0.068 | <0.001 | 0.98 | 0.996 | 0.496 |
| 35 | phenylalanine | SS | 7.45 | d | 0.11 | -0.23 | <0.001 | 9.04 | 0.90 | 0.76 | 0.009 | <0.001 | 0.043 | 0.907 | 0.888 | 0.524 | 0.29 |
| 36 | formic acid | SS | 8.48 | s | 0.09 | -0.21 | 0.0073 | 4.90 | 0.83 | 0.66 | 0.032 | <0.001 | 0.103 | 0.759 | 0.934 | 0.276 | 0.36 |
| 37 | bin-isolucine-leucine | BINS | 0.98 | s | 0.17 | 0.11 | 0.91 | 0.14 | 1.01 | 1.01 | 0.900 | 0.092 | <0.001 | <0.001 | 0.972 | 0.996 | 0.595 |
| 38 | bin-propanoic acid | BINS | 1.10 | t | 0.18 | 0.12 | 0.75 | 0.35 | 1.01 | 0.98 | 0.900 | 0.013 | 0.006 | 0.002 | 0.958 | 0.996 | 0.306 |
| 39 | bin –(LP (CC***H_2_***C=C), cadaverine, arginine, 2-hydroxybutyric acid,lysine) | BINS | 1.74 | m | 0.19 | 0.08 | 0.26 | 1.40 | 1.01 | 0.97 | 0.96 | <0.001 | 0.027 | <0.001 | 0.972 | 0.996 | 0.496 |
| 40 | bin-(LP (CC***H_2_***C=C), isovaleric acid, proline) | BINS | 2.02 | m | 0.19 | 0.11 | 0.13 | 2.11 | 0.99 | 0.89 | 0.903 | <0.001 | 0.088 | 0.016 | 0.98 | 0.996 | 0.36 |
| 41 | bin-(isovaleric acid, glutamic acid, proline) | BINS | 2.10 | m | 0.19 | 0.14 | 0.081 | 2.57 | 1.03 | 0.95 | 0.96 | <0.001 | 0.065 | <0.001 | 0.888 | 0.996 | 0.496 |
| 42 | bin-(LP (C***H_2_***CO), acetone) | BINS | 2.25 | m | 0.18 | 0.12 | 0.24 | 1.49 | 1.09 | 0.90 | 0.900 | <0.001 | 0.007 | 0.016 | 0.98 | 0.996 | 0.484 |
| 43 | bin-(3-hydroxybutyric acid) | BINS | 2.30 | m | 0.17 | 0.05 | 0.029 | 3.58 | 0.97 | 0.92 | 0.584 | <0.001 | 0.288 | 0.026 | 0.888 | 0.996 | 0.36 |
| 44 | bin-(3-hydroxybutyric acid) **^m^** | BINS | 2.42 | m | 0.02 | 0.23 | 0.015 | 4.30 | 1.08 | 1.10 | 0.076 | 0.268 | 0.425 | 0.196 | 0.612 | 0.996 | 0.35 |
| 45 | bin-glutamine | BINS | 2.47 | m | 0.02 | 0.01 | 0.37 | 1.00 | 1.00 | 1.03 | 0.960 | 0.911 | 0.024 | 0.774 | 0.888 | 0.996 | 0.271 |
| 46 | bin-(LP (C=CC***H_2_***C=C), aspartic acid | BINS | 2.76 | m | 0.16 | 0.17 | 0.40 | 0.91 | 1.01 | 0.94 | 0.960 | 0.022 | 0.108 | 0.016 | 0.934 | 0.996 | 0.36 |
| 47 | bin-(dimethylgycine, asparagine) **^m^** | BINS | 2.94 | m | -0.07 | 0.22 | <0.001 | 9.91 | 1.12 | 1.33 | 0.004 | <0.001 | 0.399 | 0.933 | 0.888 | 0.996 | 0.988 |
| 48 | bin-asparagine-albumin lysyl **^m^** | BINS | 2.98 | d | 0.01 | 0.23 | <0.001 | 7.30 | 1.06 | 1.08 | 0.002 | 0.367 | 0.597 | 0.104 | 0.612 | 0.524 | 0.484 |
| 49 | bin-tyrosine-cysteine-lysine-1-methylhistidine | BINS | 3.05 | m | 0.14 | 0.09 | 0.43 | 0.83 | 1.00 | 0.99 | 0.900 | 0.229 | 0.09 | 0.002 | 0.897 | 0.996 | 0.595 |
| 50 | bin-creatinine | BINS | 3.06 | s | 0.09 | 0.10 | 0.14 | 2.07 | 0.97 | 1.01 | 0.844 | 0.326 | 0.001 | 0.013 | 0.888 | 0.601 | 0.976 |
| 51 | bin-choline **^m^** | BINS | 3.17 | s | -0.03 | 0.26 | <0.00121 | 8.23 | 1.04 | 1.11 | 0.002 | 0.002 | 0.748 | 0.449 | 0.888 | 0.539 | 0.742 |
| 52 | bin-glucose-tyramine-histidine-TMAO-betaine-arginine-taurine | BINS | 3.25 | m | 0.17 | -0.15 | 0.0025 | 5.81 | 0.97 | 0.92 | 0.024 | <0.001 | 0.001 | 0.166 | 0.913 | 0.996 | 0.595 |
| 53 | bin-proline | BINS | 3.37 | m | 0.12 | -0.16 | 0.038 | 3.24 | 0.97 | 0.89 | 0.024 | <0.001 | <0.001 | 0.155 | 0.888 | 0.346 | 0.289 |
| 54 | bin -taurine-carnitine-glucose-proline-glutamic acid | BINS | 3.44 | m | 0.17 | -0.13 | 0.029 | 3.61 | 0.96 | 0.92 | 0.286 | <0.001 | 0.008 | 0.159 | 0.888 | 0.996 | 0.360 |
| 55 | bin-glucose-myo-Inositol | BINS | 3.55 | dd | 0.17 | -0.04 | 0.17 | 1.86 | 0.99 | 0.95 | 0.844 | <0.001 | 0.142 | 0.009 | 0.888 | 0.996 | 0.976 |
| 56 | bin-glucose-dimethylglycine-2aminobutyric acid-glutamic acid-lysine-arginine-glutamine-alanine | BINS | 3.75 | m | 0.17 | -0.04 | 0.17 | 1.82 | 0.98 | 0.96 | 0.724 | <0.001 | 0.042 | 0.014 | 0.888 | 0.996 | 0.696 |
| 57 | bin-glucose-tyrosine-creatine | BINS | 3.91 | m | 0.17 | -0.01 | 0.036 | 3.34 | 0.98 | 0.94 | 0.609 | <0.001 | 0.515 | 0.001 | 0.972 | 0.996 | 0.484 |
| 58 | bin-serine-unknown **^m^** | BINS | 3.96 | m | 0.02 | 0.24 | 0.0016 | 6.28 | 1.04 | 1.09 | 0.005 | 0.108 | 0.554 | 0.001 | 0.612 | 0.972 | 0.271 |
| 59 | bin-1-methylhistidine-phenylalanine-asparagine-serine **^f^** | BINS | 4.00 | m | 0.15 | -0.04 | <0.001 | 6.91 | 0.98 | 0.91 | 0.194 | <0.001 | 0.084 | 0.016 | 0.888 | 0.996 | 0.36 |
| 60 | bin-(LP (C***H_2_***OCOR), threonine) | BINS | 4.28 | m | 0.18 | 0.11 | 0.28 | 1.30 | 1.03 | 0.94 | 0.900 | <0.001 | 0.037 | 0.014 | 0.888 | 0.996 | 0.484 |

PCA, principal component analysis; PC, principal component; LP, lipoprotein.

^#^SS = signature signals; BINS = signals of complex intervals representing many overlapped and/or shifted signals.

*Multiplicity: s = singlet, d = doublet, t = triplet, q = quadruplet, dd = doublet of doublet, m = multiplet, s (d) = singlet of doublet, s (dd) = singlet of a doublet of doublet, s (t) = singlet of triplet, s (q) = singlet of quadruplet, d (t) = doublet of triplet, t (q) = triplet of quadruplet.

***p*-values are generated from ANOVA and corrected for the false discovery rate (FDR-p-value < 0.05). Fold change (FC) was calculated on the mean abundances between the two sheep meat intake frequency (SMIF) groups (high versus zero, and medium versus zero). eff: effect size.

****p*-values are generated from multiple linear models and corrected for the false discovery rate (FDR-p-value < 0.1). Significant metabolites for SMIF in ^m^males and in ^f^females in multiple linear models, respectively.
