# Supplementary material for: Associations between sheep meat intake frequency and blood plasma levels of metabolites and lipoproteins in healthy Uzbek adults
Source: Metabolomics. 2023 Apr 26;19(5):46. doi: 10.1007/s11306-023-02005-x (PMC10133350; doi:10.1007/s11306-023-02005-x)
Supplement: Supplementary file 2 — Supplementary file2 (DOCX 46 KB) [file 11306_2023_2005_MOESM2_ESM.docx]

Table S2. Human blood plasma lipoprotein table generated from ^1^H NMR Carr–Purcell–Meiboom–Gill (CPMG) spectra.

| N | Metabolite name | Loadings of PCA model | | SMF* | | | | SMF** | Age** | Sex** | BMI** | Total meat*** | Fish*** | Nationality  *** |
| --- | --- | --- | --- | --- | --- | --- | --- | --- | --- | --- | --- | --- | --- | --- |
|  |  | PC 1 | PC 2 | FDR-p-value | % eff | FC H/Z | FC M/Z | FDR-p-value | FDR-p-value | FDR-p-value | FDR-p-value | FDR-p-value | FDR-p-value | FDR-p-value |
| 1 | Plasma *tg* | 0.12 | -0.13 | 0.120 | 1.75 | 0.88 | 0.86 | 0.984 | <0.001 | 0.014 | 0.031 | 0.929 | 0.995 | 0.434 |
| 2 | MainFrac *tg* | 0.13 | -0.12 | 0.140 | 1.63 | 0.85 | 0.85 | 0.984 | <0.001 | 0.027 | 0.037 | 0.929 | 0.995 | 0.364 |
| 3 | SubFrac *tg* | 0.13 | -0.12 | 0.180 | 1.39 | 0.87 | 0.86 | 0.908 | <0.001 | 0.041 | 0.038 | 0.929 | 0.995 | 0.402 |
| 4 | LDL *tg* | 0.13 | -0.08 | 0.084 | 2.10 | 0.90 | 0.90 | 0.963 | <0.001 | 0.663 | 0.032 | 0.929 | 0.995 | 0.263 |
| 5 | VLDL *tg* | 0.12 | -0.13 | 0.140 | 1.64 | 0.85 | 0.83 | 0.984 | <0.001 | 0.004 | 0.032 | 0.929 | 0.995 | 0.446 |
| 6 | IDL *tg* | 0.12 | -0.08 | 0.150 | 1.55 | 0.89 | 0.88 | 0.908 | 0.001 | 0.296 | 0.032 | 0.929 | 0.995 | 0.478 |
| 7 | LDL-1 *tg* | 0.10 | -0.01 | 0.670 | 0.31 | 0.93 | 0.96 | 0.854 | 0.053 | 0.048 | 0.111 | 0.929 | 0.995 | 0.306 |
| 8 | HDL-2b *tg* | 0.05 | 0.05 | 0.470 | 0.61 | 0.90 | 1.01 | 0.903 | 0.057 | 0.186 | 0.256 | 0.934 | 0.995 | 0.992 |
| 9 | HDL-2a *tg* **^f^** | 0.04 | -0.03 | 0.120 | 1.80 | 1.16 | 1.14 | 0.139 | 0.395 | 0.972 | 0.603 | 0.934 | 0.995 | 0.807 |
| 10 | Plasma *chol* **^f^** | 0.16 | 0.02 | <0.001 | 7.53 | 0.81 | 0.88 | 0.135 | <0.001 | 0.012 | 0.075 | 0.929 | 0.995 | 0.066 |
| 11 | MainFrac *chol* | 0.16 | 0.02 | <0.001 | 8.23 | 0.80 | 0.88 | 0.138 | 0.002 | 0.284 | 0.72 | 0.934 | 0.995 | 0.069 |
| 12 | SubFrac *chol* | 0.16 | 0.01 | <0.001 | 7.73 | 0.81 | 0.88 | 0.236 | <0.001 | 0.972 | 0.256 | 0.929 | 0.995 | 0.066 |
| 13 | LDL *chol* | 0.15 | -0.02 | 0.002 | 5.44 | 0.82 | 0.87 | 0.335 | <0.001 | 0.583 | 0.103 | 0.929 | 0.995 | 0.073 |
| 14 | HDL *chol* | 0.07 | 0.24 | <0.001 | 6.79 | 0.77 | 0.93 | 0.210 | <0.001 | 0.702 | 0.096 | 0.929 | 0.995 | 0.066 |
| 15 | VLDL *chol* | 0.12 | -0.14 | 0.260 | 1.07 | 0.87 | 0.85 | 0.908 | <0.001 | 0.012 | 0.037 | 0.929 | 0.995 | 0.434 |
| 16 | IDL *chol* | 0.13 | -0.10 | 0.410 | 0.72 | 0.90 | 0.90 | 0.908 | <0.001 | 0.805 | 0.019 | 0.929 | 0.995 | 0.263 |
| 17 | LDL-1 *chol* | 0.11 | 0 | 0.072 | 2.24 | 0.87 | 0.90 | 0.984 | <0.001 | 0.003 | 0.771 | 0.929 | 0.995 | 0.446 |
| 18 | LDL-2 *chol* | 0.11 | 0.04 | 0.003 | 4.88 | 0.81 | 0.87 | 0.738 | <0.001 | 0.004 | 0.868 | 0.934 | 0.995 | 0.097 |
| 19 | LDL-3 *chol* | 0.15 | -0.03 | 0.009 | 4.03 | 0.82 | 0.87 | 0.547 | <0.001 | 0.916 | 0.061 | 0.929 | 0.995 | 0.073 |
| 20 | LDL-4 *chol* | 0.15 | -0.03 | <0.001 | 7.88 | 0.76 | 0.83 | 0.139 | 0.030 | 0.572 | 0.974 | 0.958 | 0.995 | 0.12 |
| 21 | LDL-5 *chol* | 0.15 | -0.07 | 0.002 | 5.20 | 0.79 | 0.85 | 0.245 | <0.001 | 0.068 | 0.031 | 0.929 | 0.995 | 0.073 |
| 22 | HDL-2b *chol* | 0.06 | 0.26 | 0.003 | 5.09 | 0.75 | 0.94 | 0.161 | <0.001 | 0.538 | 0.119 | 0.929 | 0.995 | 0.066 |
| 23 | HDL-2a *chol* | 0.07 | 0.24 | 0.004 | 4.76 | 0.87 | 0.95 | 0.265 | 0.036 | 0.038 | 0.474 | 0.979 | 0.995 | 0.097 |
| 24 | HDL-3 *chol* | 0.08 | 0.02 | 0.14 | 1.61 | 0.94 | 0.94 | 0.265 | 0.147 | 0.002 | 0.032 | 0.929 | 0.995 | 0.98 |
| 25 | Plasma *fchol* | 0.16 | 0.02 | 0.002 | 5.57 | 0.86 | 0.92 | 0.438 | <0.001 | 0.120 | 0.250 | 0.929 | 0.995 | 0.073 |
| 26 | VLDL *fchol* | 0.11 | -0.16 | 0.290 | 0.99 | 0.93 | 0.88 | 0.812 | <0.001 | 0.014 | 0.032 | 0.929 | 0.995 | 0.77 |
| 27 | HDL-2b *fchol* | 0.03 | 0.26 | 0.024 | 3.27 | 0.85 | 0.99 | 0.638 | 0.258 | <0.001 | 0.010 | 0.929 | 0.995 | 0.381 |
| 28 | Plasma *phosl* | 0.16 | 0.06 | 0.001 | 5.75 | 0.85 | 0.91 | 0.331 | <0.001 | 0.527 | 0.085 | 0.929 | 0.995 | 0.066 |
| 29 | MainFrac *phosl* | 0.15 | 0.06 | 0.001 | 6.00 | 0.83 | 0.90 | 0.268 | <0.001 | 0.899 | 0.103 | 0.936 | 0.995 | 0.066 |
| 30 | SubFrac *phosl* | 0.16 | 0.06 | 0.001 | 5.71 | 0.80 | 0.89 | 0.331 | <0.001 | 0.538 | 0.163 | 0.929 | 0.995 | 0.066 |
| 31 | LDL *phosl* | 0.14 | 0 | 0.001 | 5.68 | 0.81 | 0.87 | 0.286 | <0.001 | 0.348 | 0.069 | 0.929 | 0.995 | 0.068 |
| 32 | HDL *phosl* | 0.07 | 0.25 | 0.025 | 3.14 | 0.88 | 0.98 | 0.547 | 0.201 | 0.004 | 0.603 | 0.929 | 0.995 | 0.156 |
| 33 | VLDL *phosl* | 0.13 | -0.13 | 0.054 | 2.48 | 0.80 | 0.80 | 0.839 | <0.001 | 0.009 | 0.032 | 0.929 | 0.995 | 0.331 |
| 34 | LDL-1 *phosl* | 0.12 | 0.04 | 0.089 | 2.04 | 0.84 | 0.91 | 0.966 | <0.001 | 0.002 | 0.891 | 0.929 | 0.995 | 0.263 |
| 35 | LDL-2 *phosl* | 0.12 | 0.06 | <0.001 | 8.55 | 0.66 | 0.77 | 0.268 | <0.001 | 0.006 | 0.603 | 0.929 | 0.995 | 0.066 |
| 36 | LDL-3 *phosl* | 0.14 | -0.02 | 0.045 | 2.63 | 0.86 | 0.90 | 0.756 | <0.001 | 0.67 | 0.032 | 0.929 | 0.995 | 0.097 |
| 37 | LDL-5 *phosl* | 0.15 | -0.09 | 0.056 | 2.44 | 0.87 | 0.89 | 0.802 | <0.001 | 0.101 | 0.024 | 0.929 | 0.995 | 0.106 |
| 38 | HDL-2b *phosl* | 0.05 | 0.27 | 0.030 | 3.24 | 0.77 | 1.04 | 0.812 | 0.371 | 0.001 | 0.064 | 0.929 | 0.995 | 0.263 |
| 39 | HDL-2a *phosl* | 0.07 | 0.19 | 0.230 | 1.19 | 0.93 | 0.96 | 0.908 | 0.279 | 0.076 | 0.38 | 0.958 | 0.995 | 0.194 |
| 40 | Plasma *apoA1* | 0.10 | 0.19 | 0.001 | 6.06 | 0.87 | 0.95 | 0.154 | <0.001 | 0.056 | 0.063 | 0.929 | 0.995 | 0.066 |
| 41 | MainFrac *apoA1* | 0.10 | 0.20 | <0.001 | 8.23 | 0.81 | 0.91 | 0.154 | 0.010 | 0.183 | 0.931 | 0.934 | 0.995 | 0.066 |
| 42 | SubFrac *apoA1* | 0.10 | 0.19 | <0.001 | 8.45 | 0.78 | 0.90 | 0.265 | 0.004 | 0.062 | 0.047 | 0.929 | 0.995 | 0.073 |
| 43 | HDL *apoA1* | 0.10 | 0.20 | <0.001 | 8.40 | 0.82 | 0.92 | 0.210 | <0.001 | 0.695 | 0.096 | 0.929 | 0.995 | 0.066 |
| 44 | HDL-2b *apoA1* | 0.05 | 0.26 | 0.021 | 3.51 | 0.76 | 0.97 | 0.636 | 0.161 | 0.002 | 0.037 | 0.929 | 0.995 | 0.306 |
| 45 | HDL-2a *apoA1* | 0.08 | 0.21 | <0.001 | 6.34 | 0.82 | 0.92 | 0.139 | 0.030 | 0.043 | 0.111 | 0.97 | 0.995 | 0.178 |
| 46 | HDL-3 *apoA1* **^f^** | 0.12 | 0.05 | <0.001 | 6.89 | 0.83 | 0.89 | 0.139 | <0.001 | 0.452 | 0.04 | 0.929 | 0.995 | 0.066 |
| 47 | Plasma *apoB* | 0.16 | -0.04 | <0.001 | 6.45 | 0.83 | 0.88 | 0.265 | <0.001 | 0.908 | 0.037 | 0.929 | 0.995 | 0.066 |
| 48 | MainFrac *apoB* | 0.16 | -0.05 | 0.002 | 5.41 | 0.83 | 0.88 | 0.331 | <0.001 | 0.916 | 0.044 | 0.929 | 0.995 | 0.066 |
| 49 | SubFrac *apoB* | 0.16 | -0.04 | 0.001 | 5.72 | 0.81 | 0.87 | 0.319 | <0.001 | 0.916 | 0.073 | 0.929 | 0.995 | 0.066 |
| 50 | LDL *apoB* | 0.15 | -0.04 | 0.001 | 5.89 | 0.84 | 0.89 | 0.268 | <0.001 | 0.805 | 0.037 | 0.929 | 0.995 | 0.09 |
| 51 | VLDL *apoB* | 0.13 | -0.12 | 0.009 | 4.04 | 0.78 | 0.84 | 0.322 | <0.001 | 0.111 | 0.044 | 0.934 | 0.995 | 0.397 |
| 52 | IDL *apoB* | 0.13 | -0.08 | 0.030 | 2.98 | 0.87 | 0.89 | 0.665 | <0.001 | 0.916 | 0.019 | 0.929 | 0.995 | 0.178 |
| 53 | LDL-1 *apoB* | 0.10 | 0.01 | 0.11 | 1.86 | 0.90 | 0.92 | 0.961 | 0.001 | 0.002 | 0.818 | 0.958 | 0.995 | 0.389 |
| 54 | LDL-2 *apoB* | 0.11 | 0.04 | 0.002 | 5.32 | 0.81 | 0.87 | 0.737 | <0.001 | 0.001 | 0.426 | 0.976 | 0.995 | 0.092 |
| 55 | LDL-3 *apoB* | 0.15 | -0.05 | 0.012 | 3.75 | 0.83 | 0.88 | 0.689 | <0.001 | 0.992 | 0.064 | 0.929 | 0.995 | 0.073 |
| 56 | LDL-4 *apoB* | 0.15 | -0.05 | 0.002 | 5.34 | 0.79 | 0.85 | 0.275 | <0.001 | 0.482 | 0.037 | 0.929 | 0.995 | 0.066 |
| 57 | LDL-5 *apoB* | 0.15 | -0.06 | 0.001 | 5.85 | 0.75 | 0.84 | 0.138 | <0.001 | 0.228 | 0.600 | 0.929 | 0.995 | 0.068 |
| 58 | LDL-6 *apoB* | 0.08 | -0.11 | 0.17 | 1.50 | 1.06 | 0.86 | 0.268 | 0.009 | 0.024 | 0.073 | 0.929 | 0.995 | 0.992 |
| 59 | Plasma *chole* | 0.16 | 0.02 | <0.001 | 7.41 | 0.81 | 0.87 | 0.210 | <0.001 | 0.908 | 0.085 | 0.929 | 0.995 | 0.066 |
| 60 | VLDL *chole* | 0.13 | -0.13 | 0.035 | 2.84 | 0.78 | 0.80 | 0.756 | <0.001 | 0.014 | 0.036 | 0.929 | 0.995 | 0.263 |
| 61 | IDL *chole* | 0.15 | -0.09 | 0.005 | 4.54 | 0.77 | 0.81 | 0.380 | <0.001 | 0.643 | 0.010 | 0.929 | 0.995 | 0.073 |
| 62 | LDL *chole* | 0.15 | -0.03 | 0.002 | 5.18 | 0.79 | 0.84 | 0.541 | <0.001 | 0.979 | 0.110 | 0.929 | 0.995 | 0.066 |
| 63 | LDL-1 *chole* | 0.12 | 0.03 | 0.014 | 3.70 | 0.78 | 0.86 | 0.966 | <0.001 | 0.001 | 0.447 | 0.929 | 0.995 | 0.263 |
| 64 | LDL-3 *chole* | 0.14 | 0 | <0.001 | 7.34 | 0.74 | 0.82 | 0.138 | <0.001 | 0.348 | 0.771 | 0.929 | 0.995 | 0.066 |
| 65 | HDL-2b *chole* | 0.08 | 0.22 | 0.001 | 6.69 | 0.67 | 0.87 | 0.249 | 0.232 | 0.002 | 0.103 | 0.929 | 0.995 | 0.263 |

PCA, principal component analysis; PC, principal component; *tg*, triglycerides; eff, effect size; FC, fold change; H/Z, the ratio of lipoprotein in high sheep meat intake frequency (SMIF) group to in zero SMIF group; M/Z, the ratio of lipoprotein in moderate sheep meat intake frequency (SMIF) group to in zero SMIF group; FDR-p-value, false discovery rate (FDR) corrected p-values; chol, cholesterol; fchol, free cholesterol; phosl, phospholipids; LDL, low-density lipoprotein; HDL, high-density lipoprotein; phosl, phospholipid; Mainfrac, main fraction; Subfrac, subfraction; ApoA1, apolipoprotein A1; ApoB, apolipoprotein B; chole, cholesterol ester.

**p*-values are generated from ANOVA and corrected for the false discovery rate (FDR-p-value < 0.05). Fold change was calculated on the mean abundances between the two sheep meat intake frequency (SMF) groups (high versus zero, and medium versus zero). eff: effect size.

***p*-values are generated from multiple linear models and corrected for the false discovery rate (FDR-p-value < 0.1). Significant metabolites for SMF in ^f^females in multiple linear models, respectively.
